# Early assessment of response to induction therapy in acute myeloid leukemia using ^18^F-FLT PET/CT

**DOI:** 10.1186/s13550-017-0326-8

**Published:** 2017-09-16

**Authors:** Eun Ji Han, Bo-hee Lee, Jeong-A Kim, Young Ha Park, Woo Hee Choi

**Affiliations:** 1 Department of Radiology, Daejeon St. Mary’s Hospital, College of Medicine, The Catholic University of Korea, 64, Daeheung-ro, Jung-gu, Daejeon, 34943 South Korea; 2 Divison of hematooncology, Department of Internal Medicine, G Sam Hospital, Hyosan Medical Foundation, 591, Gunpo-ro, Gunpo, Gyeonggi-do 15839 South Korea; 3 Department of Hematology, St. Vincent’s Hospital, College of Medicine, The Catholic University of Korea, 93, Jungbu-daero, Paldal-gu, Suwon, Geonggi-do 16247 South Korea; 4 Department of Radiology, St. Vincent’s Hospital, College of Medicine, The Catholic University of Korea, 93, Jungbu-daero, Paldal-gu, Suwon, Geonggi-do 16247 South Korea

**Keywords:** Acute myeloid leukemia, Bone marrow, ^18^F-FLT, Induction therapy, PET, Response assessment

## Abstract

**Background:**

We evaluated the suitability of ^18^F-fluorodeoxythymidine (^18^F-FLT) positron emission tomography (PET)/computed tomography (CT) for assessment of the early response to induction therapy and its value for predicting clinical outcome in patients with acute myeloid leukemia (AML). Adult patients who had histologically confirmed AML and received induction therapy were enrolled. All patients underwent ^18^F-FLT PET/CT after completion of induction. PET/CT images were visually and quantitatively assessed. Cases with intensely increased bone marrow uptake in more than one third of the long bones and throughout the central skeleton were interpreted as PET-positive for resistant disease (RD). PET results were compared to the clinical response and outcome.

**Results:**

In visual PET analysis of 10 eligible patients (7 male, 3 female; median age 58 years), 5 patients were interpreted as being PET-positive and 5 as PET-negative. Standardized uptake values were significantly different between PET-positive and PET-negative groups. Eight of 10 patients achieved clinical complete remission (CR)/CR with incomplete blood count recovery (CRi). Five CR/CRi patients had PET-negative findings, but 3 CR patients had PET-positive findings. Both of the RD patients had PET-positive findings. During follow-up, 2 CR patients with PET-positive findings relapsed, or were strongly suspected of relapse, 4 months after consolidation.

**Conclusion:**

^18^F-FLT PET/CT after induction therapy showed good sensitivity and negative-predictive value for evaluating RD in patients with AML. This preliminary study suggests that ^18^F-FLT PET/CT may be valuable as a noninvasive tool for early assessment of the response to treatment and may provide prognostic value for survival in patients with AML.

## Background

Acute myeloid leukemia (AML) is a biologically and clinically heterogeneous disease with a distinct response to treatment and clinical outcome. AML treatment typically entails induction chemotherapy aimed at debulking disease and rapidly restoring hematopoiesis, followed by consolidation therapy to eliminate residual disease. Only a few predictive factors are used to make treatment decisions in clinical practice, although the prognosis of patients with AML is influenced by several patient-specific and disease-related risk factors, among which cytogenetic risk is most important [1].

Early assessment of the response to induction therapy is important for defining chemosensitivity and for planning subsequent treatment and can be valuable in predicting prognosis in patients with AML. Previous studies have reported that the early response to induction therapy in AML is an independent predictor of subsequent complete remission (CR) and is associated with the long-term outcome [2, 3]. The current National Comprehensive Cancer Network and European LeukemiaNet guidelines recommend an early bone marrow (BM) evaluation, at 14−21 days after the start of induction therapy, to assess the early response to treatment and to guide further treatment [4]. Recently, however, the interim BM assessment has come into question. Systematic study has never been tested for the value of interim BM assessment. The predictive value of the interim BM assessment for achieving CR varies in sensitivity (40−90%) and specificity (43−79%) [5–8]. Not all patients with negative interim BM results achieve CR, while some patients with significant blast numbers in the interim BM show recovered counts and documented CR without undergoing further therapy. Furthermore, the lack of standardized criteria for the optimal response or residual/refractory disease makes it more challenging to use interim BM results to guide further management. BM aspiration is generally regarded as safe, but invasive [9, 10]. Most patients report pain during BM aspiration, with 36% grading this pain as moderate to severe; this causes stress and anxiety for patients and their families. Additionally, invasive procedures are associated with risks of bleeding and infection in the BM nadir after induction, although the complication rate is low [9, 10].

^18^F-fluorodeoxythymidine (^18^F-FLT) is a radiopharmaceutical for positron emission tomography (PET) that reflects cell proliferation. ^18^F-FLT is trapped after phosphorylation by thymidine kinase1 (TK-1), whose expression is increased in replicating cells [11]. Because ^18^F-FLT uptake is specific for cycling cells, ^18^F-FLT PET could be used in hematologic diseases featuring BM abnormalities. Agool et al. [12, 13] have demonstrated that ^18^F-FLT PET imaging can be used to evaluate and quantify BM proliferation and is useful for distinguishing hematologic disorders. This study aimed to evaluate whether ^18^F-FLT PET/computed tomography (CT) is suitable for assessment of the early response to induction chemotherapy and is helpful for predicting prognosis in patients with AML.

## Methods

### Patients

This study was performed in accordance with the approved guidelines of our hospital’s institutional review board. Written consent was obtained from all participants included in the study.

Adult patients with histologically confirmed AML, diagnosed between September 2014 and October 2015, were included in this prospective study. Patients with acute promyelocytic leukemia were excluded. All patients underwent BM aspiration with cytogenetic assessment; molecular genetic analysis of FLT3 and NPM1 was performed in 7 patients. All BM samples were interpreted according to the World Health Organization Classification of Tumors of Hematopoietic and Lymphoid tissues [14]. Risk stratification, based on cytogenetics and molecular abnormalities, was used to categorize patients into three groups: favorable-, intermediate-, and unfavorable-risk groups [4]. Clinical and histological information, such as age, sex, Eastern Cooperative Oncology Group performance status, complete blood count, lactate dehydrogenase (LDH) titer, and the findings of BM aspiration at diagnosis, and survival outcomes were obtained from medical records.

All patients received induction chemotherapy with the intent of achieving CR. Patients aged < 60 years were treated with cytarabine and daunorubicin in the standard “7 + 3” regimen, and patients aged ≥ 60 years were treated with cytarabine and idarubicin. All patients underwent interim ^18^F-FLT PET/CT imaging 7−12 days after commencing induction therapy (Table 1).

**Table 1:** Characteristics of ^﻿18^F-FLT PET/CT studies

Patient no.	Time of ^18^F﻿-FLT PET/CT study	Weight (kg)	Injected activity^a^ (MBq)	Time interval from injection to imaging (min)
1	Day 8	58	170.2	66
2	Day 10	55	177.6	58
3	Day 9	55	155.4	61
4	Day 7	59	199.8	59
5	Day 8	69	266.4	79
6	Day 8	69	281.2	58
7	Day 9	44	210.9	57
8	Day 10	80	240.5	71
9	Day 9	62	273.8	74
10	Day 12	55	262.7	78

^a^The injected activity was calculated by subtracting the post-injection syringe activity from loaded syringe activity before injection

### ^18^F-FLT PET/CT imaging

Imaging for all patients was obtained using integrated PET/CT scanner (Gemini TF; Philips, Best, The Netherlands). ^18^F-FLT (2.96 MBq/kg) in 2−5 mL of normal saline was injected intravenously. One hour after ^18^F-FLT injection, CT commenced from the vertex or orbitomeatal line and progressed down toward the upper thigh using a standard protocol, 120 kVp, 100 mA, and 4-mm slice thickness. PET data were then acquired immediately for 1 min per bed position. PET images were reconstructed using 3D ordered subset iterative time-of-flight reconstruction technique (BLOB-OS-TF) with 3 iterations, 33 subsets, 144 × 144 matrix in 234 slices, and voxel sizes of 4 × 4 × 4 mm^3^. The spatial resolution of the images was about 4.3 mm. All activities were corrected for decay of ^18^F-FLT from the start of the PET scanning back to the time of ^18^F-FLT injection.

### Image analysis

PET/CT images were analyzed on a workstation with fusion software (version 6.3, MIM Software Inc., Cleveland, OH, USA). Two nuclear medicine physicians who were blinded to the BM result reviewed the PET/CT images to consensus. All PET/CT images were visually interpreted for the intensity of BM uptake and the degree of BM expansion and were classified as PET-positive or PET-negative. A PET-positive result, consistent with resistant disease (RD), was defined as the presence of intensely increased BM uptake involving most of the central skeleton and one third or more along the length of the long bones, such as bilateral humeri and femurs. Visual assessment could readily distinguish between PET-positive and PET-negative groups, because the normal distribution of ^18^F-FLT is limited in the central skeleton and ultra-proximal humeri and femurs [12, 15]. The mean standardized uptake value (SUV) of ^18^F-FLT uptake was measured at the intertrochanteric area of the bilateral femurs, posterior crest of the bilateral iliac bones, bodies of lumbar vertebra 4 (L4), thoracic vertebra 12 (T12) and thoracic vertebra 6 (T6), and the sternum. The SUV is the mean value of a 1.2-cm-diameter volumetric region of interest (ROI) within the marrow space. Additionally, the SUVs of the liver and spleen were measured by drawing a 3-cm spherical ROI. For evaluation of BM distribution, the ROI was defined to include the cervical, thoracic, and lumbar vertebrae from the CT images, and this ROI was copied and applied to the PET images. From the PET images, only the voxels with ^18^F-FLT uptake values greater than the background were considered to correspond to the marrow, and thus, voxels with values greater than or equal to 0.5 were included in the PET ROI [15]. From the PET ROI, SUVs and their standard deviation (SD) were measured.

### Assessment of clinical response

Patients underwent a follow-up BM aspiration 4−6 weeks after induction chemotherapy to evaluate the clinical response. Treatment response was defined according to International Working Group for Diagnosis, Standardization of Response Criteria, Treatment Outcomes and Reporting Standards for Therapeutic Trials in Acute Myeloid Leukemia 2003 criteria [16]. After the end of the treatment period, the patients were followed up at regular intervals. The following tests were performed at follow-ups: physical examination, complete blood count with differential, blood chemistry, and BM biopsy, if clinically indicated.

### Statistical analysis

Categorical variables were expressed as an absolute number and continuous variables were expressed as median or mean ± SD and range. The heterogeneity of BM SUV distribution was calculated based on the coefficient of variation (CV), which is the SD normalized by the mean. The Mann−Whitney *U* test was used to compare the SUVs between PET-positive and PET-negative groups. Time to relapse was defined as the time from the date of induction therapy to the date of detected relapse or last clinical follow-up. Follow-up time was defined as the time from the date of induction therapy to the date of death or last clinical follow-up. All statistical analyses were performed using the Statistical Package for the Social Sciences software (IBM Corp., Armonk, NY, USA). *P* values < 0.05 were considered statistically significant.

## Results

### Patient characteristics

Ten eligible patients with AML were enrolled (7 male, 3 female; median age, 58 years). Of these, 1 patient (patient 4) was found to have secondary AML, evolved from myelodysplastic syndrome (refractory anemia with excess blasts-2) and 1 patient (patient 3) developed second relapsed AML with a history of achieving CR after intensive chemotherapy 4 years earlier. Based on cytogenetic and molecular abnormalities, 3 patients were classified as having favorable-risk disease, 5 as having intermediate-risk disease, and 2 as having poor-risk disease. Three patients had a low white blood cell (WBC) count (< 4 × 10^9^/L) and 7 patients had an elevated LDH titer (> 230 U/L; Table 2).

**Table 2:** Patient characteristics

Patient no.	Age	Sex	ECOG PS	Risk status	BM blasts (%)	BM cellularity (%)	WBC (× 10^9^/L)	Hb (g/dL)	Platelet (× 10^9^/L)	LDH (U/L)
1	60	M	1	Poor	52	52	40.86	11.6	57	450
2	72	M	1	Indeterminate	30	30–40	3.23	4.7	27	378
3	40	F	1	Indeterminate	8	90	2.65	9.8	191	136
4	48	F	1	Indeterminate	33	80–90	2.1	7.2	112	124
5	66	M	0	Indeterminate	45	50	13.3	9.6	50	216
6	36	F	1	Favorable	21	80	17.34	7	10	571
7	73	M	2	Poor	39	100	19.02	4.6	10	606
8	19	M	1	Indeterminate	68	100	21.43	4.8	35	825
9	60	M	3	Favorable	25	80	30.72	3	11	706
10	55	M	2	Favorable	26	80	14.18	8.4	56	1144

*ECOG PS* Eastern Cooperative Oncology Group performance status, *Hb* hemoglobin

### ^18^F-FLT PET/CT findings

**Fig. 1:**
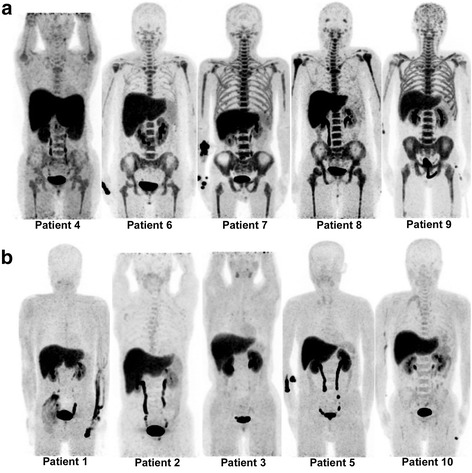
^18^F-FLT PET maximum intensity projection images after induction therapy in 10 patients with AML. Five PET cases (**a**) showed intensely increased BM uptake throughout the central skeleton and bilateral humeri and femurs and were interpreted as PET-positive. The other 5 PET cases (**b**) were interpreted as PET-negative. Of these, 2 (patients 1 and 3) had invisible BM uptake throughout the central skeleton and bilateral humeri and femurs. Three cases (patients 2, 5, and 10) demonstrated perceptible BM uptake along the central skeleton; however, the intensity of ^18^F-FLT uptake was markedly lower than that of the liver and no significant uptake was seen along bilateral humeri and femurs

The median interval between the start of induction chemotherapy and ^18^F-FLT PET/CT scanning was 9 days (range, 7−12 days). All patients tolerated the PET/CT scanning well, with no adverse reactions. In visual ^18^F-FLT PET/CT analysis, 5 patients were interpreted as being PET-positive and 5 as PET-negative (Fig. 1). Two patients (patients 4 and 6) had hepatosplenomegaly. The CVs and SUVs for all sites of each patient are presented in Table 3. The CVs of the PET-positive group were > 0.3 and only 1 patient (patient 2) in the PET-negative group had a CV of 0.32. Significant differences were observed between PET-positive and PET-negative groups for SUVs of all sites (Table 4). The mean SUV was 4.4 (range, 3.06−5.72) in the liver and 1.3 (range, 0.69−2.07) in the spleen; hepatic (*p* = 0.251) and splenic (*p* = 0.917) uptake did not differ significantly between PET-positive and PET-negative groups.

**Table 3:** Visual and quantitative analyses of ^18^F-FLT PET/CT images

Patient no.	Visual analysis	CV	Quantitative analysis (SUV)							
			Right femur	Left femur	Right iliac	Left iliac	L4	T12	T6	Sternum
1	−	0.16	0.22	0.32	0.45	0.62	0.54	0.82	0.41	0.50
2	−	0.32	0.25	0.39	0.88	0.63	0.59	1.29	1.38	0.73
3	−	0.22	0.52	0.66	0.50	0.88	0.81	0.91	0.82	0.81
4	+	0.35	1.43	1.42	1.26	1.31	1.43	1.42	1.30	1.36
5	−	0.26	0.34	0.73	0.73	0.89	0.98	1.06	0.89	0.80
6	+	0.34	2.46	2.65	2.77	2.92	3.07	3.01	2.85	2.70
7	+	0.42	2.57	2.16	2.32	2.52	3.04	3.08	3.39	2.69
8	+	0.43	3.40	3.47	3.60	4.00	3.84	3.69	3.79	4.68
9	+	0.46	3.48	3.40	4.37	3.97	4.95	4.64	4.84	4.46
10	−	0.19	0.76	0.88	1.14	1.04	1.15	1.28	1.25	1.16

**Table 4:** Differences of SUVs between the 2 groups according to visual analysis

Visual analysis	Quantitative analysis (mean SUV ± SD)							
	Right femur	Left femur	Right iliac	Left iliac	L4	T12	T6	Sternum
PET-positive group	2.67 ± 0.83	2.62 ± 0.86	2.86 ± 1.19	2.94 ± 1.12	3.27 ± 1.29	3.17 ± 1.18	3.23 ± 1.30	3.18 ± 1.38
PET-negative group	0.42 ± 0.22	0.60 ± 0.24	0.74 ± 0.28	0.81 ± 0.18	0.81 ± 0.26	1.07 ± 0.21	0.95 ± 0.38	0.80 ± 0.24
*p* value	0.009	0.009	0.009	0.009	0.009	0.009	0.016	0.009

### Association of ^18^F-FLT uptake and clinical outcome

In the follow-up BM study, 7 of 10 patients achieved clinical CR, 1 patient had morphological CR with incomplete blood count recovery (CRi), and 2 patients had RD. During the follow-up period (median, 20 months; range, 3−34 months), 5 of the 10 patients died. The clinical outcomes of the 10 patients are presented in Table 5. Both of the RD patients had PET-positive findings. One patient (patient 4) received re-induction and then died from pneumonia at the nadir. The other patient (patient 8) underwent re-induction and consolidation and has been in remission. Among the 8 patients with CR/CRi, 5 patients had PET-negative findings, but 3 patients had PET-positive findings. During the follow-up period, relapse was confirmed or highly suspected in 3 patients. One patient (patient 2), with PET-negative findings but with a high CV, relapsed and died. Two (patient 6 and 7) of 3 patients with negative follow-up BM tests, but PET-positive findings, relapsed or were strongly suspected of relapse. Although patient 6 achieved CR after induction therapy, clinical relapse was strongly suspected due to frequent infection, severe thrombocytopenia (30 × 10^9^/L), and an elevated LDH titer (1041 U/L) at 4 months after consolidation; however, the patient was transferred to another hospital without pathological confirmation of relapse. One patient (patient 9) with PET-positive findings has been in CR but has had persistent thrombocytopenia. More clinical information of the 3 patients with discrepant findings are presented in Table 6.

**Table 5:** Clinical outcome of 10 patients

Patient no.	Visual ^18^F-FLT uptake	FU BM result	Following treatment after induction	Relapse	Time to relapse (months)	Death	Follow-up time (months)
1	−	CR	Consolidation and PBSCT	No	16	Yes^a^	16
2	−	CRi	Consolidation	Yes	7	Yes	18
3	−	CR	Consolidation	No	34	No	34
4	+	RD	Re-induction	–^b^	–^b^	Yes	3
5	−	CR	Consolidation	No	29	No	29
6	+	CR	Consolidation	Yes	–^c^	Yes	17
7	+	CR	Consolidation	Yes	10	Yes	11
8	+	RD	Re-induction and consolidation	No	23	No	23
9	+	CR	Consolidation	No	21	No	21
10	−	CR	Consolidation	No	21	No	21

*PBSCT* peripheral stem cell transplantation

^a^Patient 1 died from graft-versus-host disease

^b^Patient 4 had never achieved a CR/CRi after initial induction therapy

^c^Patient 6 had strongly suspected but unconfirmed relapse 10 months from the start of induction therapy

**Table 6:** Cytogenetics and laboratory follow-up in 3 patients with discrepant findings

		At baseline	At PET/CT imaging	At FU BM	After consolidation			
					1 month	4 months	10 months	16 months
Pt 6	Cytogenetics	46, XX, t(8;21)(q22;q22)[19]/46,XX[1]						
	WBC (× 10^9^/L)	17.34	0.68	3.41	4.47	17.16	–^a^	–^a^
	Hb (g/dL)	7	9.6	10.8	11	13.2	–^a^	–^a^
	Platelet (× 10^9^/L)	10	21	13	70	30	–^a^	–^a^
	LDH (U/L)	571	169	343	246	1041	–^a^	–^a^
Pt 7	Cytogenetics	43-45, XY, del(3)(p11.2), del(5)(q13q33),−7,+8,del(12)(p11.2p11.2), −21[cp20]						
	WBC (× 10^9^/L)	19.02	0.85	3.09	3.3	8.2	–^a^	–^a^
	Hb (g/dL)	4.6	9.1	7.7	9	9.4	–^a^	–^a^
	Platelet (× 10^9^/L)	10	32	176	170	38	–^a^	–^a^
	LDH (U/L)	606	347	229	178	418	–^a^	–^a^
Pt 9	Cytogenetics	45,X,−Y, t(8;21)(q22;q22), add(14)(q32)[20] : RUNX1-RUNX1T1						
	WBC (× 10^9^/L)	30.72	0.66	5.38	11.41	4.65	4.65	6.13
	Hb (g/dL)	3	10.3	12.5	10.5	13.7	15.4	15.2
	Platelet (× 10^9^/L)	11	55	297	49	97	97	105
	LDH (U/L)	706	310	227	306	151	153	178

*Pt* patient

^a^Patient 6 and 7 relapsed or were strongly suspected of relapse at 4 months after consolidation

## Discussion

Although BM aspiration with morphological assessment remains the standard for residual disease assessment in patients with AML, its predictive value for residual disease is suboptimal and the technique is invasive [5–8]. To complement this limitation of BM aspiration, noninvasive modalities have been applied to assess the presence of viable residual cells in BM after induction. Several studies have suggested that dynamic contrast-enhanced magnetic resonance imaging of BM can be used to assess the changes in microvascular density and could be a useful prognostic indicator of disease activity and survival [17–19]. ^18^F-FDG PET imaging, which measures glucose metabolism, has also been shown to have potential for evaluation of leukemic BM infiltration [20].

As a surrogate of cellular proliferation, ^18^F-FLT PET has been used for early therapeutic monitoring in various cancers [21–24]. The rate-limiting step for intracellular ^18^F-FLT retention in proliferating cells is phosphorylation by TK-1, and thus, ^18^F-FLT accumulates in proportion to TK-1 activity [25]. A more than 10-fold overexpression of TK-1, which is the key enzyme for intracellular ^18^F-FLT accumulation, is observed in leukemic blasts [26]. Only a few studies have reported the feasibility of ^18^F-FLT PET imaging in AML. In a pilot study by Buck et al. [27], patients with relapsed, refractory, or untreated leukemia showed higher ^18^F-FLT uptake in the BM and spleen than did controls. This indicated that ^18^F-FLT PET reflected disease activity, but there was no significant correlation between ^18^F-FLT uptake and the number of leukemic blasts identified in BM biopsy. The authors discussed that the proliferative activity of leukemic blasts and normal BM cannot be differentiated using ^18^F-FLT PET, as the normal hematopoietic cells also show increased ^18^F-FLT uptake. In another study of AML patients who underwent induction therapy, the BM uptake in RD patients was significantly greater than in normal controls, while the BM uptake in CR patients was similar to that in normal controls in pre-treatment ^18^F-FLT PET images [15]. That study also investigated whether ^18^F-FLT PET during or after induction therapy could be useful for early assessment of the clinical response. All 5 CR patients exhibited low BM uptake, while 2 RD patients displayed elevated BM uptake (maximum SUV, 3.6 ± 0.4 vs. 11.4 ± 0.8, *p* < 0.001). Thus, the study concluded that ^18^F-FLT PET during induction chemotherapy could predict successful BM ablation early on. We presumed that increased ^18^F-FLT uptake is associated with the degree of RD in the present study, because all PET/CT scans were performed shortly after myeloablative chemotherapy. Our results indicated that interim ^18^F-FLT PET/CT performed well in the early prediction of clinical response to induction therapy in patients with AML. All 5 patients with PET-negative findings achieved CR in the follow-up BM aspiration 4−6 weeks after induction therapy (high negative-predictive value), and both of the RD patients showed PET-positive findings (high sensitivity).

Leukemic distribution can be heterogeneous or localized, despite the malignant systemic disease [28, 29]. Vanderhoek et al. [15] demonstrated substantial heterogeneity of BM uptake in the ^18^F-FLT PET response assessment, both during and after induction therapy. The distribution of ^18^F-FLT uptake in RD patients was more heterogeneous (higher CV) than that in CR patients. Additionally, 1 RD patient was in an aplastic state at the interim BM biopsy, despite the significantly increased BM uptake in the ^18^F-FLT PET image. The BM biopsy is usually performed on the unilateral posterior iliac bone and the BM evaluation is thus limited to that area and cannot reflect the whole of the BM in vivo; therefore, residual leukemia might be missed. ^18^F-FLT PET imaging is performed from the vertex to the upper thigh after a single injection, facilitating noninvasive assessment of the total hematopoietic BM compartment, which represents a significant advantage over a BM biopsy.

The present study also suggested that ^18^F-FLT PET/CT may perform better than follow-up BM assessment in predicting relapse. Three patients with PET-positive findings achieved CR in the follow-up BM aspiration (false-positive). Of these, 2 patients (patients 6 and 7) had confirmed or clinically suspected relapse within 4 months after consolidation, even though 1 patient had been classified as favorable-risk. On the other hand, patient 3, with relapsed AML, had PET-negative findings and has now been in the third remission for 34 months, although relapse after achieving remission is considered a poor prognostic factor in patients with AML [30]. One of the 5 PET-negative patients relapsed; this patient showed a heterogeneous BM distribution (CV = 0.32). In a study conducted by Vanderhoek et al. [15], the CVs of all patients with residual/refractory disease on the interim BM biopsy were ≥ 3.0. BM heterogeneity as well as visual analysis may be important for predicting relapse. Current risk-adapted therapy in adult patients with AML is based on only a few prognostic markers, such as age, cytogenetic risk, and gene mutations at diagnosis. More recently, adjustment of therapy based on incorporation of post-treatment data is likely to become increasingly important. When added to the current risk-stratification system, ^18^F-FLT PET/CT may provide a more accurate selection of patients at high risk for relapse, and thus improve survival by modification of treatment.

In our CR patients, 1 false-positive PET case (patient 9) showed persistent mild thrombocytopenia (Table 6). Agool et al. [12] reported that significantly increased ^18^F-FLT uptake was observed in patients with myelodysplasia, myeloproliferative disorder, and myelofibrosis. Although our patient was not further evaluated for thrombocytopenia, there was a possibility that BM dysfunction caused the false-positive PET finding.

The main limitation of this study is the small sample size. Additionally, molecular studies were not available in all patients. Risk stratification, based on combined clinical and molecular markers, has recently been proposed to improve the predictive value of early response assessment [1, 4]. Our results suggest that further studies on the prognostic potential of the combination of molecular abnormalities and interim ^18^F-FLT PET/CT results are warranted. The heterogeneity of compliance to the imaging protocol is another potential limitation. We originally planned to standardize the imaging protocol strictly. However, variability in the injected activity and in the time from injection to PET scanning was unavoidable, and some cases had accidental extravasation at the injection site. Poor protocol compliance may lead to over- or underestimation, particularly in terms of quantitative PET analysis. To overcome this limitation, we performed both qualitative and quantitative analyses. In qualitative analysis, we experienced no difficult cases when determining whether the image was positive or negative according to our criteria. For solid cancers that are already frequently and usefully evaluated with PET/CT, qualitative PET analysis is as important as quantitative PET analysis. For lymphoma, the Deauville criteria is based on visual analysis [31]. Lastly, there were neither standards in place regarding the time for the ^18^F-FLT PET/CT imaging of patients with AML nor any established criteria for interpreting the images. The role of imaging studies, including PET/CT, is still limited in terms of evaluation of hematologic malignancy. However, application of ^18^F-FLT PET/CT to patients with AML has been continuously studied although the number is small [15, 27, 32]. Recently, a phase II clinical trial assessing ^18^F-FLT PET/CT in AML was launched [32], and this prospective ECOG-ACRIN EAI141 is likely to provide more conclusive results when completed. Our preliminary results could be of value in planning future clinical studies (e.g., determining appropriate time points for performing ^18^F-FLT PET/CT), data analysis (e.g., setting the criteria for visual analysis), and the clinical interpretation of ^18^F-FLT PET/CT images in patients with AML. We anticipate that these study results will collectively contribute to the establishment of clinical applications of ^18^F-FLT PET/CT imaging in patients with AML.

## Conclusion

The results of this preliminary study showed good sensitivity and negative-predictive value for evaluating residual disease in patients with AML by means of ^18^F-FLT PET/CT, although unexpected discrepant findings were noted in 3 of 10 cases, possibly reflecting the heterogeneous nature of AML. Our results suggest that ^18^F-FLT PET/CT may have value as a noninvasive tool for early assessment of the response to treatment and may provide prognostic value for survival in patients with AML.

## Abbreviations

^18^F-FLT: ^18^F-fluorodeoxythymidine
AML: Acute myeloid leukemia
BM: Bone marrow
CR: Complete remission
CRi: CR with incomplete blood count recovery
CT: Computed tomography
CV: Coefficient of variation
ECOG PS: Eastern Cooperative Oncology Group performance status
Hb: Hemoglobin
L4: Lumbar vertebra 4
LDH: Lactate dehydrogenase
PBSCT: Peripheral stem cell transplantation
PET: Positron emission tomography
RD: Resistant disease
ROI: Region of interest
SD: Standard deviation
SUV: Standardized uptake value
T12: Thoracic vertebra 12
T6: Thoracic vertebra 6
TK-1: Thymidine kinase1
WBC: White blood cell
